# Antibacterial Properties of Quercetin–Curcumin–Modified PMMA Denture Base Resin for Oral Surgical Site Applications: An In Vitro Study

**DOI:** 10.1155/ijod/9600760

**Published:** 2026-04-15

**Authors:** Anjali Varghese, Aindrias Ryan, Abdurahman Salem, Chibuzo Nlemorisa

**Affiliations:** ^1^ Department of Dental Technology, Queens Dental Sciences Centre, University of Greater Manchester, Bolton, UK; ^2^ Department of Microbiology, Centre for Clinical and Biomedical Sciences, University of Greater Manchester, Bolton, UK

**Keywords:** antibacterial activity, curcumin, dental biomaterials, oral surgical site infection, PMMA resin, quercetin

## Abstract

Surgical site infections (SSIs) remain a significant complication following oral and maxillofacial surgeries, largely due to bacterial adhesion on prosthetic materials, causing infections and delayed wound healing. Quercetin and Curcumin which are natural polyphenols have been reported to possess antioxidant and antibacterial potentials. This study investigated the antibacterial effects of incorporating a quercetin–curcumin (QC) complex into heat‐cured polymethyl methacrylate (PMMA) resins against *Staphylococcus aureus* and *Pseudomonas aeruginosa* in vitro. Disc‐shaped PMMA specimens (8 mm × 2 mm) were fabricated with neat PMMA and 2% and 3% QC complex at 3:1 ratio. Antibacterial activity was assessed by initially using disc diffusion to optimize the QC concentration, followed by colony‐forming unit (CFU) counts with QC incorporated into the PMMA. Statistical analysis was performed using Kruskal–Wallis tests, with significance set at *p*  < 0.05. The disc diffusion assay revealed inhibition although not statistically significant (*p*  > 0.05) with increasing QC concentration. At 3 wt% QC, inhibition zones reached 8 mm for *S. aureus* and 2 mm for *P. aeruginosa*, however, no inhibition in the negative controls. CFU counts showed a fluctuating trend with *S. aureus* viability declining at 2 wt% of QC as opposed to that of *P. aeruginosa* which showed a consistent decrease in viability with increasing QC concentration even though this difference not statistically significant (*p*  > 0.05). A 3 wt% QC in PMMA exhibited encouraging antibacterial activity, particularly against *P. aeruginosa*, suggesting its potential utility for prosthetic rehabilitation in oral and maxillofacial surgery; however, additional studies are required to confirm its efficacy and clinical applicability.

## 1. Introduction

Surgical site infections (SSIs) remain a significant postoperative complication in oral and maxillofacial surgery [1]. Their occurrence is influenced by several factors including surgical invasiveness, perioperative contamination, and the presence of oral microbial flora that may colonize biomaterials placed immediately after surgery, such as obturators or surgical prostheses [2]. SSIs, the major precursor of nosocomial infections constitute about 20% of infections acquired postsurgery in Rezaei et al. [3]. In head and neck oncological cases, the incidence can exceed 20%, significantly impairing wound healing and patient outcomes [4, 5].

The oral cavity presents a particularly high risk for SSIs due to its dense microbial flora and favorable conditions for biofilm development. The oral cavity provides a favorable environment for microbial colonization because of its diverse microbiota and constant exposure to moisture and nutrients. Opportunistic pathogens such as *Staphylococcus aureus* and *Pseudomonas aeruginosa* are capable of adhering to biomaterial surfaces and forming structured biofilms, which enhances bacterial persistence and resistance to host immune responses and antimicrobial agents [6]. Biofilm‐associated bacteria especially the staphylococci have proved more resistant to antimicrobials compared with planktonic forms, compounding the clinical challenge of tackling bacterial infections [7, 8].

Traditional strategies for infection control, including systemic antibiotics and antiseptic mouth rinses, provide limited protection. As such, antimicrobial sutures and topical antibiotic applications have been explored [2], but these methods are transient and risk adverse effects such as toxicity or disruption of the oral microbiome. Since the global health has been challenged by increasing development of antimicrobial‐resistant strains due to antibiotic overuse and misuse, it has become imperative to re‐engineer the existing antibiotics or develop new ones [9].

Polymethyl methacrylate (PMMA) remains one of the most widely used denture base materials in prosthetic dentistry due to its ease of processing, acceptable biocompatibility, esthetic properties, and relatively low manufacturing cost [10]. However, PMMA is biologically inert and provides no intrinsic defense against microbial colonization [11], risking denture‐induced stomatitis and secondary infections due to surface‐level microbial adherence. In recent years, efforts to reduce or prevent pathogenic oral diseases such as periodontitis, dental caries, and endodontic infections have focused on incorporating bioactive agents into dental restorative materials to impart therapeutic functionality [12].

Numerous strategies have been explored to impart antimicrobial functionality to PMMA–based dental materials. Previous studies have incorporated agents such as metallic nanoparticles, antimicrobial monomers, peptides, and phytochemical compounds into the resin matrix to inhibit microbial colonization [13–16]. Although these approaches can be effective, some metallic additives raise concerns related to ion release, oxidative stress [17], and long‐term biocompatibility, prompting increasing interest in safer and naturally derived antimicrobial alternatives.

Plant‐derived polyphenolic compounds have attracted attention as potential antimicrobial agents in biomedical materials [15]. Quercetin, a flavonoid commonly found in fruits and vegetables, possesses antioxidant, anti‐inflammatory, and antimicrobial properties [18, 19]. Similarly, curcumin, a polyphenolic compound obtained from the rhizome of *Curcuma longa*, chemically known as 1,7‐bis(4‐hydroxy‐3‐methoxyphenyl)‐1,6‐heptadiene‐3,5‐dione, has demonstrated broad‐spectrum antimicrobial activity against bacteria, fungi, and viruses [20, 21]. It exhibits multifunctional biological activities comparable to quercetin and has been widely utilized in traditional medicine for the treatment and management of various medical conditions [14, 20], and in extension, for the prevention and treatment of dental caries and oral disinfection [22, 23]. The demonstrated efficacy of curcumin in eradicating pathogenic oral microbiomes, such as *Streptococcus mutans*, supports its suitability for oral applications.

When combined, these compounds may exert synergistic antimicrobial effects through complementary mechanisms in which quercetin disrupts bacterial membranes and interfere with nucleic acid synthesis, while curcumin inhibit cellular division—activities which simultaneously promote wound‐healing [24–26].

The synergistic potential of incorporating quercetin and curcumin into dental biomaterials although proven, has remained underexplored. In the effort to prevent SSIs and mitigate the rise of antimicrobial resistance, investigating the antibacterial efficacy of quercetin–curcumin (QC) incorporated into PMMA resin for use in surgical applications is imperative. In light of the growing need for antimicrobial dental biomaterials, this study investigated the antibacterial potential of incorporating a QC complex into heat‐cured PMMA denture base resin. The antibacterial activity of the modified material was evaluated against *S. aureus* and *P. aeruginosa*, two pathogens commonly implicated in postoperative infections.

## 2. Materials and Methods

### 2.1. Specimen Preparation

**Figure 1:**
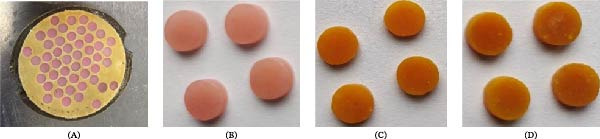
(A) Wax discs invested in a stone mold, (B) QC0, (C) QC2, and (D) QC3 PMMA discs.

Nine disc‐shaped (8 mm × 2 mm) PMMA specimens (*n* = 3/group) were fabricated using lost‐wax technique and processed by conventional heat‐curing method (Figure 1). QC ratio of 3:1 was chosen as it is proven to be the most biocompatible, however, with human dermal fibroblasts [25]. The control group was prepared by mixing Ivoclar polymer and monomer (Ivoclar Vivadent, Schaan, Liechtenstein) according to the manufacturer’s instructions (2.5 g:1 mL) and was designated as QC 0% (QC0). For the experimental groups, a 3:1 quercetin and curcumin (Sigma–Aldrich, St. Louis, MO, USA) complex was prepared by measuring and manually mixing both compounds using a ceramic motar and pestle, and afterwards, a metal spatula until homogeneity was achieved. A calculated amounts (2% *w*/*w* and 3% *w*/*w* of the PMMA powder weight) of this complex was then added into the corresponding volume of the monomer in a glass beaker. A 3% *w*/*w* QC was adapted from Khanal et al. [27] and Khamooshi et al. [28], and modified. This was left to mix on a magnetic stirrer at 300 rpm under room temperature for 30 min until visually uniform suspension was achieved. Subsequently, the corresponding quantity of PMMA powder was added to the QC–monomer phase and spatulated and allowed to reach stage before packing into the prepared molds (Figure 1) and curing at 100°C for 2 h in a curing unit (Labormat SD, Dreve, Germany). This procedure was replicated for the 3 wt% group. The 2% *w*/*w* and 3% *w*/*w* formulations were designated QC2 and QC3, respectively. After polymerization, the specimens were polished through 500, 1000, and 1200 grit SiC paper and finally polishing cloth with paste using a metallographic grinder (Tegramin 25, Struers, France). Prior to testing, all discs were washed twice in distilled water and subsequently immersed in 70% ethanol for 1 min before air drying in a sterile condition [29].

### 2.2. Bacteria Culture

*Staphylococcus aureus* (ATCC 9144) and *P*. *aeruginosa* (ATCC 27853) were cultured using the streak plate method. For *S. aureus*, a 10 µL disposable loop was used to transfer the bacterial suspension onto Mueller–Hinton agar (MHA) plates (Oxoid, United Kingdom, CM0337B). The plates were incubated in an inverted position at 37°C for 24 h. A single colony was subsequently isolated and inoculated into 5 mL of tryptic soy broth (Oxoid, UK) in a universal container, mixed using a vortex mixer (Classic Vortex Mixer, Fisherbrand, Leicestershire, UK), and incubated overnight at 37°C with agitation at 200 rpm in an orbital shaker incubator (ES‐20, Grand‐Bio, Cambridge, UK). For *P. aeruginosa*, a high‐density inoculum was prepared following ATCC’s recommendation by adding 0.5 mL of bacterial suspension to 6 mL of TSB. A streak plate was prepared using 10 µL of this suspension and incubated overnight at 37°C. A single colony was then transferred to nutrient broth, vortexed, and incubated overnight at 37°C to obtain the overnight culture. Following this, 1/100 dilution of each overnight culture was prepared by inoculating 100 µL of bacterial suspension into 9.9 mL of TSB supplemented with 0.25% glucose.

### 2.3. Preparation of Agar Plates and Media

MHA was prepared according to the manufacturer’s instructions (38 g/L) using deionized water. The mixture was thoroughly agitated and sterilized by autoclaving at 121°C for 15 min (Labclave Autoclave, LTE Scientific Ltd, Oldham, United Kingdom). Approximately 20 mL of the sterilized agar solution was dispensed into sterile petri dishes to prepare agar plates. Tryptone soya broth (TSB) was similarly prepared following the manufacturer’s instructions (30 g/L) in deionized water and sterilized under the same autoclaving conditions described for MHA.

### 2.4. Disc Diffusion Assay

**Figure 2:**
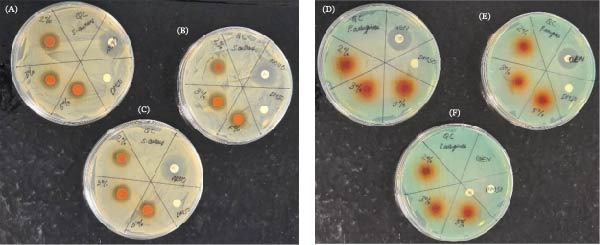
Representative triplicate agar plates showing zones of inhibition (ZOI) for (A–C) *Staphylococcus aureus* and (D–F) *Pseudomonas aeruginosa*.

The disc diffusion assay was conducted to determine the optimal concentration (2%, 3%, and 5%) at which 3:1 ratio of QC is most effective against *S. aureus* and *P. aeruginosa*. A 2% *w*/*v*, 3% *w*/*v*, and 5% *w*/*v* QC were each dissolved in 10 mL of dimethyl sulfoxide (DMSO; Fisher Scientific, Geel, Belgium). Following this, 20 µL each of the formulations were impregnated into paper discs and were allowed to air‐dry under a sterile condition. Subsequently, MHA plates were inoculated with 200 µL *of S. aureus* or *P. aeruginosa* and were spread gently with L‐shaped spreader. Novobiocin (5 µg/mL) and gentamicin (10 µg/mL) served as positive controls for *S. aureus* and *P. aeruginosa* respectively, while DMSO (10 µg/mL) discs served as a negative control (for both bacteria), to validate the assay and confirm bacterial responsiveness. Zones of inhibition (Figure 2A–F) were measured after 24 h incubation at 37°C.

### 2.5. Colony‐Forming Unit (CFU) Counts

**Figure 3:**
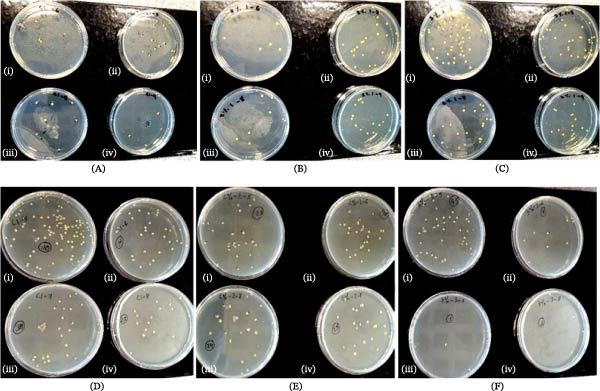
Sample agar plates showing colony‐forming unit (CFU) counts for 10^−6^–10^−9^ dilutions of (A; i–iv)—QC0; (B; i–iv)—QC2, and (C; i–iv)—QC3 for *Staphylococcus aureus*; 10^−5^–10^−8^ dilutions of (D; i–iv)—QC0; (E; i–iv)—QC2, and (F; i–iv)—QC3 for *Pseudomonas aeruginosa*.

The sterile neat PMMA and the QC–PMMA discs were transferred to 48 well plate. To each of the wells, 500 µL of diluted overnight bacterial culture was added to each well and was mildly shaken for even distribution of the bacteria before incubating for 48 h at 37°C. Following this, the wells were gently washed with clean water and were allowed to dry for about 15 min. A 500 µL of phosphate‐buffered saline (PBS; Dulbecco’s PBS, Corning, Manassas, USA) was then added to each well followed by sonication in an ultrasonic water bath 150 W 50‐Hz frequency (Ultra sonic cleaning bath, Walkers electronics limited, Newark, Nottinghamshire, UK) for 6 min to disrupt the biofilm. A 1/1000 serial dilution was carried out by adding 900 µL of PBS to designated wells in 24 well plates (TC Plate 24 Well, Sarstedt, England). Experiment was conducted in triplicates (*n* = 3) [29]. To each of these wells, 100 µL bacterial media from the ultrasonicated wells was added to the 1st well and serially diluted up to the 9th well for each specimen of all groups. Spread plate culture for the *S. aureus* was prepared from the 10^−5^ to 10^−8^ dilution and 10^−6^–10^−9^ for the *P. aeruginosa* by transferring and spreading 100 µL of the respective dilutions on nutrient agar plates followed by incubation at room temperature for 24 h. Experiment was conducted in triplicates and bacterial colonies (Figure 3A–F) counted and expressed as CFU/mL.

### 2.6. Statistical Analysis

Data were analyzed using IBM SPSS Statistics for Windows, version 28. Normality of data was tested using Shapiro–Wilk test followed by Kruskal–Wallis and a pairwise comparison among groups, with Bonferroni correction and *p*  < 0.05. Prior to statistical analysis, CFU/mL data were log‐transformed to normalize data distribution and stabilize variance.

## 3. Results

### 3.1. Disc Diffusion Assay

**Figure 4:**
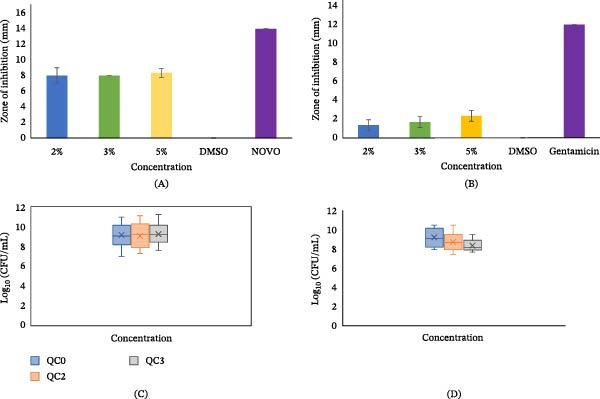
Mean (SD) of the zone of inhibition for (A) *Staphylococcus aureus* and (B) *Pseudomonas aeruginosa* and log_10_ (CFU/mL) counts for (C) *S*. *aureus* and (D) *P*. *aeruginosa*.

The mean (SD) and percentage inhibition for both *S. aureus* and *P. aeruginosa* are presented in Table 1 with Figure 2A,B showing zones of inhibition on the agar plates, and Figure 4A,B, the graphs representing the ZOI data. There was a statistically significant difference in the zones of inhibition with both bacteria (*p*  < 0.05). QC2 and QC3 both demonstrated a 57.14% inhibition against the *S. aureus*, and QC5 59.28%. In contrast, a lower inhibition, QC2 (11.03%), QC3 (13.83%), and QC5 (19.41%) were observed with the *P. aeruginosa*.

**Table 1:** Mean and standard deviation of the zones of inhibition for *Staphylococcus aureus* and *Pseudomonas aeruginosa* at 24 h.

Concentration	*Staphylococcus aureus*		*Pseudomonas aeruginosa*	
	Mean ± standard deviation (mm)	Percentage of inhibition (%)	Mean ± standard deviation (mm)	Percentage inhibition (%)
DMSO	0	0	0	0
QC2	8 ± 1	57.14	1.33 ± 0.57	11.03
QC3	8	57.14	1.66 ± 0.57	13.83
QC5	8.3 ± 0.57	59.28	2.33 ± 0.57	19.41
(Novobiocin/gentamicin)	14	100	12	100

### 3.2. CFU Counts

The mean and standard deviation were back‐transformed and reported in CFU/mL for interpretability and presented in Table 2. Figure 3A–F illustrates the corresponding CFU counts of *S. aureus* and *P. aeruginosa* on representative agar plates. Figure 4C,D shows the box and whisker plots of log_10_ CFU/mL values illustrating the central tendency and variability. For *S. aureus*, CFU counts exhibited a fluctuating trend with the concentrations; however, the differences among groups were not statistically significant (*p*  > 0.05). The highest count was observed in the control group, QC0 (1.06 × 10^10^ ± 16.93 CFU/mL), followed by QC3 (1.38 × 10^9^ ± 13.14 CFU/mL) and QC2 (9.35 × 10^8^ ± 20.57 CFU/mL). In contrast, *P. aeruginosa* exhibited a decreasing trend in CFU counts with increasing QC concentration, although a pairwise comparison between QC3 and QC0 showed a significant difference (*p*  < 0.05), the overall among the groups was not statistically significant (*p*  > 0.05). The QC3 group showed the lowest viable count (1.78 × 10^8^ ± 4.03 CFU/mL), followed by QC2 (5.50 × 10^8^ ± 10.82 CFU/mL), while QC0 recorded the highest count (1.10 × 10^9^ ± 9.04 CFU/mL).

**Table 2:** Back‐transformed mean (SD) of the CFU counts for *Staphylococcus aureus* and *Pseudomonas aeruginosa* at 24 h.

Concentration	*Staphylococcus aureus*	*Pseudomonas aeruginosa*
	Mean ± standard deviation (CFU/mL)	Mean ± standard deviation (CFU/mL)
QC0	1.06 × 10^10^ ± 16.93	1.10 × 10^9^ ± 9.04
QC2	9.35 × 10^8^ ± 20.57	5.50 × 10^8^ ± 10.82
QC3	1.38 × 10^9^ ± 13.14	1.78 × 10^8^ ± 4.03

## 4. Discussion

Immediate prosthetic devices used following oral and maxillofacial surgery often remain in close contact with surgical wounds, creating a potential surface for microbial colonization. Effective preoperative decolonization strategies have been shown to reduce SSIs, which is particularly important given the increasing spread of resistant pathogens such as methicillin‐resistant *S*. *aureus* (MRSA) [30]. In the present study, the disc diffusion assay demonstrated inhibition zones of approximately 8 mm for the 2% and 3% QC groups against *S*. *aureus* and approximately 2 mm against *P*. *aeruginosa*, while no inhibition was observed in the DMSO control. The positive controls, novobiocin and gentamicin, produced inhibition zones of up to 14 and 12 mm against *S. aureus* and *P. aeruginosa*, respectively, validating the experimental model. CFU analysis further revealed a dose‐dependent reduction in *P. aeruginosa* viability, whereas *S. aureus* demonstrated a less consistent response. Nevertheless, the QC formulations demonstrated measurable antibacterial activity in the diffusion assays, consistent with previous in vitro reports [25, 31]. Although the antimicrobial effects observed were modest and varied between bacterial species, the modified resin demonstrated reduced bacterial viability, particularly for *P*. *aeruginosa*. Similar moderate antibacterial responses have been reported in early‐stage investigations of phytochemical‐modified dental polymers prior to optimization of concentration and release behavior [14].

The greater reduction in *P. aeruginosa* viability may reflect the susceptibility of this gram‐negative pathogen to plant‐derived polyphenols. Quercetin has been reported to disrupt quorum sensing pathways, increase membrane permeability, and inhibit nucleic acid synthesis and biofilm formation in *P. aeruginosa* [24, 32]. Curcumin has similarly been shown to inhibit bacterial motility and cell division, resulting in membrane disruption while also interfering with bacterial adhesion and biofilm formation in oral pathogens such as *Streptococcus mutans* [20, 22].

In addition to antibacterial activity, quercetin possesses strong antioxidant and radical‐scavenging properties, including the ability to bind transition metal ions and neutralize reactive oxygen species (ROS) [25, 33]. These properties may influence the biological behavior of PMMA–based biomaterials. Similar to antioxidant additives such as N‐acetyl cysteine and mannitol, quercetin may reduce oxidative stress associated with PMMA polymerization and potentially limit cell necrosis in surrounding tissues [34–36]. This observation is supported by Alavi et al. [37], who demonstrated that quercetin can reduce the cytotoxic effects of silver nanoparticles through phenolic interactions and antioxidant activity. The protective effect is thought to depend on the ratio between quercetin and curcumin, with enhanced biological performance reported at higher quercetin‐dominant formulations [25].

Overall, incorporation of 3% QC into PMMA demonstrated slightly greater antibacterial activity compared with the 2% formulation. Although both concentrations produced similar inhibition zones (~8 mm against *S. aureus* and ~2 mm against *P. aeruginosa*), CFU analysis indicated improved suppression of *P. aeruginosa* viability at higher QC concentrations (Table 2 and Figure 3A–F). These findings support previous reports demonstrating enhanced bactericidal efficacy when quercetin and curcumin are combined due to their complementary antimicrobial mechanisms [25, 38].

Interestingly, while *P. aeruginosa* demonstrated progressive reduction in viability with increasing QC concentration, *S. aureus* exhibited a reduction at 2% QC followed by a relative increase at 3%. This nonlinear response may be related to the limited aqueous solubility of quercetin and curcumin, which can lead to molecular aggregation at higher concentrations and reduce the availability of active antimicrobial molecules [39]. Furthermore, higher concentrations of phytochemicals may induce bacterial adaptive responses such as stress tolerance or biofilm formation, which are well‐recognized survival mechanisms in *S. aureus* populations [20, 31, 40].

Numerous strategies have been explored to impart antimicrobial properties to PMMA denture base materials, including incorporation of metallic nanoparticles, antimicrobial monomers, phytochemicals, and antibiotics [13, 14, 16]. Although, there is lack of specific comparative studies between the potency of QC and inorganic antimicrobial agents, metallic nanoparticles such as silver, copper, and titanium have demonstrated strong antimicrobial activity against oral pathogens [13]. However, concerns regarding cytotoxicity, oxidative stress, and ion release remain important limitations associated with metallic nanoparticle‐modified biomaterials [17]. Compared with conventional antiseptics such as chlorhexidine (CHX), which provide rapid broad‐spectrum antimicrobial activity, phytochemicals such as curcumin and quercetin offer advantages in terms of improved biocompatibility and sustainability for biomaterial applications [23]. On the perspective of synergy, curcumin and quercetin can demonstrate a combinatorial antimicrobial effect with other antimicrobial agents such as nisin or silver nanoparticles in acrylic resins [14, 37].

In addition to antimicrobial effects, curcumin‐based additives have been reported to contribute to acceptable mechanical properties in acrylic resins due to interactions between phenolic hydroxyl groups and the polymer matrix, allowing them to function as both fillers and bioactive additives [14, 27, 41, 42].

From a clinical perspective, antimicrobial PMMA materials may reduce microbial colonization on prosthetic surfaces and potentially lower the risk of postoperative infections. This is particularly relevant for patients undergoing head and neck cancer resection, reconstructive surgery, or trauma repair, who often require immediate prosthetic rehabilitation and may be predisposed to infection due to prolonged hospitalization and compromised immune status [4, 5]. Although the findings in this study are modest, they lay a foundation for future investigations that may be translated into clinical approaches to potentially reduce postoperative infections, limit reliance on systemic antibiotics, and help mitigate the spread of antimicrobial‐resistant pathogens in surgical environments.

Several limitations are acknowledged. The relatively small sample size may limit the statistical strength of the findings, and the study did not evaluate biofilm formation, which is a critical factor in biomaterial‐associated infections. Additionally, the release kinetics and diffusion behavior of quercetin and curcumin from the PMMA matrix were not investigated, which may influence the magnitude and duration of antibacterial activity. Although both compounds have demonstrated favorable biological profiles in previous studies, cytotoxicity testing using oral fibroblast cell lines is required to confirm the safety of QC–modified PMMA.

Another limitation relates to the poor aqueous solubility of quercetin and curcumin, which may restrict their release from the PMMA matrix. However, nano‐formulations or conjugation with nanoparticles may enhance solubility, diffusion, and bioavailability, thereby improving antimicrobial efficacy compared with bulk forms [39, 43].

Future studies should therefore investigate the polymerization behavior, physicomechanical properties, and long‐term antibacterial performance of QC–modified PMMA to ensure that antimicrobial enhancement does not compromise structural integrity. Evaluation of flexural strength, surface hardness, and impact resistance will be particularly important. Further research should also assess release kinetics, long‐term antibacterial stability, and cytocompatibility with oral fibroblasts and keratinocytes [44]. In addition, exploring synergistic combinations of QC with other natural or synthetic antimicrobial agents may further enhance antibacterial efficacy [32, 45]. Such investigations will be essential to validate the clinical applicability of phytochemical‐modified denture base materials and support the development of more sustainable antimicrobial biomaterials.

## 5. Conclusion

Within the limitations of this preliminary in vitro study, incorporation of a QC complex into PMMA demonstrated modest antibacterial effects, particularly against *P*. *aeruginosa*. Although these findings suggest potential for developing phytochemical‐modified denture materials, further studies investigating biofilm inhibition, compound release kinetics, mechanical properties, and cytotoxicity are necessary before clinical applicability can be considered. These findings highlight the potential for developing antimicrobial prosthetic materials that may reduce SSIs following oral and maxillofacial surgery, laying a foundation to incorporating natural plant‐derived antimicrobials, a promising step towards a safer and more sustainable dental biomaterials reengineering.

## Author Contributions

**Anjali Varghese, Chibuzo Nlemorisa, and Aindrias Ryan:** investigation, methodology, formal analysis, visualization. **Anjali Varghese, Chibuzo Nlemorisa, Aindrias Ryan, and Abdurahman Salem**: writing – original draft, writing – review and editing. **Chibuzo Nlemorisa and Abdurahman Salem**: conceptualization, project supervision.

## Funding

This study was not funded by any agency in the public, commercial, or nonprofit sectors.

## Disclosure

All authors have read and approved the final manuscript.

## Ethics Statement

This study was approved by the Research Ethics Committee at the University of Bolton (Now Greater Manchester; Certificate Number AUTONOHP00588). All methods were performed following the Declaration and the ethical guidelines of the University of Bolton Research Ethics Committee. No human participants or tissue were involved in the study.

## Conflicts of Interest

The authors declare no conflicts of interest.

## Data Availability

The datasets generated and analyzed during the current study are available from the corresponding author upon reasonable request.
